# Experimentally induced mood changes and simultaneously changing heart rate variability (HRV)

**DOI:** 10.1080/21642850.2026.2709754

**Published:** 2026-09-03

**Authors:** Maud Vroemen, Anne Marsman, Thérèse van Amelsvoort, Richel Lousberg

**Affiliations:** a Department of Psychiatry and Neuropsychology, Maastricht University, Maastricht, the Netherlands; b Stichting Traumanet, De Meern, the Netherlands

**Keywords:** Heart rate variability, mood induction procedure, ECG, emotions, emotional valence

## Abstract

**Objective:**

To investigate the relationship between rapidly changing emotional states and simultaneously measured heart rate variability (HRV).

**Methods:**

Eighty-two healthy subjects underwent a mood induction procedure while ECG recordings were made. The subjects were asked to look at an image for 30 s and then instructed to visualize the image again with closed eyes for 1 min. RR intervals were calculated as the time between two adjacent R-peaks. HRV was calculated by taking the square root of the mean absolute differences between RR intervals for each 10-s segment. This value was then logarithmically transformed.

**Results:**

Following a series of validation analyses, the relationship between mood changes and HRV was assessed. A first positively loaded image corresponded with higher levels of HRV compared to a first negatively loaded image. For the second image of opposite emotional valence, no reversal of HRV could be demonstrated. In contrast, the HRV level measured during the first image continued during the visualization phase of the second image.

**Conclusion:**

This study provides evidence that experimentally manipulated mood covaries with HRV. However, when mood states change rapidly, the relationship with HRV is not detectable. Several post-hoc explanations are provided. Possible implications for clinical practice are discussed.

## 1. Introduction

‘Cogito, ergo sum’. With this famous dictum, René Descartes became one of the founders of modern philosophy. Descartes distinguished two entities: the res cogitans, also referred to as the mental substance or the soul, and the res extensa, often translated as corporeal substance. Although Descartes described this dualism, he also suggested an evident interaction between the mind and body. He partly explained this interaction through the pineal gland, which he defined as the seat of the soul. Thus, the mind-body problem, as we know it today, was formulated (2022). mind-body dualism |. Definition, Theories, & Facts | Britannica. Accessed. https://www.britannica.com/topic/mind-body-dualism).

Descartes described emotions as the ‘passions of the soul’ because ‘of all the kinds of thought which the soul may have, there are none that agitate and disturb it so strongly’ (Cottingham et al., 1985). The definition of emotions was later extensively discussed by William James in his identically titled book (James, 1884). Both philosophers, as well as physician Lange, argued that emotional experiences are the consequences of physiological reactions to stimuli (Kirkebøen, 2019; Lang, 1994). However, this James-Lange theory was later challenged by the Cannon-Bard theory, which proposed that emotional experience can occur simultaneously with, and independently from, physiological arousal (D’Hondt et al., 2010).

Current research on emotional experience and the origin of emotions remains influenced by this longstanding debate. Yet, testing these theories and disentangling causal interactions between the mind and body in experimental settings is challenging. Advances in our understanding of the anatomical and functional characteristics of the brain, supported by imaging techniques such as fMRI, have provided greater insight into the complex network organization involved in emotional experience (Pessoa, 2017). However, imaging studies often lack participants’ subjective emotional experience. An alternative approach to illuminate this debate is therefore the use of self-reported mood in combination with biomarkers. Biomarkers are defined as ‘a characteristic that is measured as an indicator of normal biological processes, pathogenic processes, or responses to an exposure or intervention’ (Silver Spring (MD): Food & Drug Administration (US); Bethesda (MD): National Institutes of Health (US), 2022). Their scope can range from diagnostic or prospective to epigenetic or metabolomic (Califf, 2018; García-Gutiérrez et al., 2020).

Against this background, the present study addresses a more specific and currently underexplored question: whether HRV is sensitive not only to relatively stable emotional or clinical states, but also to rapidly changing emotional states that unfold within a short experimental time frame. In daily life, emotional experiences often change quickly, for example, when a pleasant event is followed by disappointing news or when a stressful situation is rapidly resolved. These real-life emotional dynamics are difficult to capture with longer HRV measurement windows, which may average out short-lived psychophysiological responses. By combining a sequential mood induction procedure with ECG recordings segmented into ultra-short intervals, the present study aims to explore whether the HRV corresponds with initial and subsequent changes in emotional valence.

An emerging parameter and biomarker of interest in the psychophysiological research field is heart rate variability (HRV). HRV, defined as the variation in the time interval between adjacent heartbeats, is useful for gaining more insight into the functioning of the autonomic nervous system (ANS) (Acharya et al., 2026). Regarding healthy individuals, researchers have reported that HRV correlates with better general health and that higher levels of HRV are associated with emotional well-being (Acharya et al., 2006; Mather & Thayer, 2018; Ogliari et al., 2015). Individuals with higher HRV are better at regulating their emotions and score better on neuropsychological performances as well. Cattaneo and colleagues concluded that people with lower levels of HRV tend to experience emotional dysregulation (Cattaneo et al., 2021). Additionally, these individuals need a longer period to recover from psychological stressors compared to individuals with higher levels of HRV.

Regarding psychiatric diagnoses, recent literature shows HRV is used as an indicator of emotional control in individuals with, among others, binge eating, schizophrenia, bipolar disorder, and alcohol use disorders. Concerning those who suffer from objective binge-eating episodes (OBE), interesting conclusions regarding the relationship between guilt and HRV have been reported. In a study by Bottera and colleagues among 86 participants with and without OBE (39.5% and 60.5%, respectively), a procedure to induce guilt or a neutral mood state was carried out, and the participants were then asked to consume a meal replacement shake until they were satisfied (Bottera et al., 2021). During the entire procedure, the HRV was measured. Guilt was measured at the start of the experiment, before consuming the shake, and after consuming the shake. It was concluded that, for those in the guilt condition, guilt intensity and HRV had an inverse relationship. The researchers suggest HRV can be used to reflect one's emotion regulation capabilities. They concluded that being less capable of regulating emotions is associated with more problems with guilt regulation.

In another study investigating whether HRV can be seen as a potential biomarker of disease severity in individuals with schizophrenia and bipolar disorder, 35 participants with schizophrenia, 52 participants with bipolar disorder, and 149 healthy participants were included (Benjamin et al., 2021). The results showed that individuals with schizophrenia and those with bipolar disorder had significantly lower HRV compared to healthy subjects. An extensive review of HRV in patients with alcohol use disorder showed that healthy participants had higher levels of HRV at rest compared to those with alcohol use disorder and moderate/heavy drinkers (Ralevski et al., 2019). Overall, the interpretation of HRV remains inherently context-dependent, as its broader relationship with physical health is complex; however, within the domain of emotional and psychological functioning, higher HRV seems to generally be associated with more adaptive regulation.

A conceptual distinction should therefore be made between trait-level and state-level HRV. Trait-level HRV generally refers to relatively stable interindividual differences or baseline autonomic functioning, as often examined in clinical populations or chronic conditions. State-level HRV, by contrast, concerns short-term changes in autonomic regulation that may occur in response to a specific stimulus or emotional state. Whereas much of the existing literature focuses on trait-like or resting HRV, the present study focuses on state-level dynamics by investigating whether HRV changes in relation to experimentally induced, rapidly changing emotional states.

To quantify HRV, time-domain, frequency-domain, and non-linear indices are described (Shaffer & Ginsberg, 2017). The current literature provides extensive research in which the HRV is calculated over relatively long periods, ranging from hours to sometimes even days (Goffeng et al., 2018; Reardon & Malik, 1996). Focusing on clinical populations, such as those with depression, this implies that HRV effects are related to relatively stable mood states, even though they are diminished compared to healthy individuals (Hartmann et al., 2018; Sgoifo et al., 2015). In other words, the differences in baseline HRV between healthy and depressed individuals result from small changes in HRV that eventually aggregate to a generally lower level of HRV in the clinical population. However, when assessing rapidly changing emotions, such as those experienced in daily life, HRV quantification over longer periods is inadequate. It can be concluded that longer HRV measurements can predict health outcomes, whereas relatively short measurements of HRV can be seen as ‘proxies of proxies’ (Shaffer et al., 2020). When investigating short-term HRV data in the time domain, the resulting data set yields a series of repeated measures in a nested structure (Snijders & Bosker, 2011). This means that repeated HRV segments are embedded within subjects. The preferred statistical technique for such data structures is multi-level analysis.

Considering the above, we decided to set up a new study to investigate whether short-term, state-level HRV corresponds with experimentally induced changes in mood state, with particular attention to rapid shifts in emotional valence. To do so, a recently redesigned mood induction procedure was combined with ECG recordings. In this procedure, two images of opposite emotional loading were presented subsequently to 82 healthy participants (Vroemen et al., 2023). The results of this mood induction experiment showed participants reported greater changes in mood after exposure to the second image. Even though the effect of the first image, contrasted with the baseline mood, seems to be a less biased estimation of a change in the HRV, the contrast in the subjective mood is highest between image 1 and image 2. Additionally, it is worth mentioning that a previous pilot study showed it is necessary to make at least 10-s ECG recordings to quantify a relatively stable HRV estimate. Therefore, segments of 10 s were incorporated into the design of this study. Since associations between the HRV and startle reflex can be found, the potential effects of blinking on the presented images were minimized by analysing the HRV during a closed-eyes condition only (Jaén et al., 2021; Ruiz-Padial et al., 2003).

Finally, since the effects of colour and recognition of the images cannot be ignored in the validation of mood studies, these two variables were included as potential confounders in the current study (Vroemen et al., 2023).

Before testing the key experimental hypotheses, three basic, validating hypotheses were formulated based on findings in the current literature. The first hypothesis addresses the natural inverse relationship between heart rate (HR) and HRV: a higher HR is associated with a lower HRV (Sacha, 2014; Tsuji et al., 1996). The reasoning behind this phenomenon can be found in the autonomic nervous system: as HR increases, there is less time for the RR interval to vary. The second hypothesis suggests that older adults tend to have a lower HRV due to a decreased ability of the autonomic nervous system to react to external stimuli (de Meersman & Stein, 2007; Fukusaki et al., 2000; Reardon & Malik, 1996). The third hypothesis proposes that HRV is lower in women compared to men, according to an extensive meta-analysis by Koenig and Thayer (Koenig & Thayer, 2016). These results imply that women’s greater mean HR and lower HRV might be due to parasympathetic dominance, whereas men might have a sympathetically dominant heart. Nevertheless, it should also be mentioned that this sex-dependent mechanism is complex, with several questions still to be answered (Ryan et al., 1994).

In sum, the three validating hypotheses are:

1. A higher HR is correlated with lower levels of HRV.
2. In older adults, a lower level of HRV is expected compared to their younger counterparts.
3. HRV levels are expected to be lower in women compared to men.
  The next two hypotheses focus on the central theme of this article, namely, to investigate the effects of the mood induction procedure on HRV:
4. The HRV level measured during a positive mood induction is higher than the HRV level measured during a negative mood induction.
5. Given an initial mood induction in either a positive or a negative direction accompanied by congruent HRV levels, a subsequent mood induction in the opposite direction results in a congruent change in HRV.

## 2. Materials and methods

### 2.1. Participants

Potential participants were recruited via flyers distributed in the city of Maastricht, the Netherlands. The exclusion criteria were: (1) structural use of antipsychotics, antidepressants, antiepileptics, and anxiolytics during the past year; (2) structural use of alcohol in excess of >5 units/day during the past year; (3) visual or hearing disability; and (4) illiteracy or dyslexia. Alcohol consumption in the evening before the experiment was discouraged, as well as consuming caffeine 3 h before the experiment began. Written informed consent was obtained from all participants. The participants who completed all the experiments in the more extensive study were rewarded with €50. Ultimately, data from 82 participants were available.

### 2.2. Materials

A mood induction procedure, which included two images to initiate a positive mood induction and two images to induce a negative mood induction, was carried out (Vroemen et al., 2023). The participants were exposed to either a positive image first and a negative image second or vice versa. Three items to grade participants’ happiness, sadness, and anger were assessed before the mood induction procedure, after seeing the first image, and after seeing the second image. Happiness, sadness, and anger were graded on a Likert scale from 0 to 6, where 0 represented ‘not at all’ and 6 represented ‘very much’.

Since the mood induction procedure is described elsewhere by Vroemen and colleagues, here the essentials of the procedure are summarized (Vroemen et al., 2023). During this mood induction procedure, ECG recordings were made with a BrainAmp ExG amplifier (Brain Products). To sample the data at 1000 Hz, the Brain Vision Recorder software was used.

### 2.3. Procedure

The participant was placed opposite a computer screen in an electrically and sound-shielded room. To register the ECG recordings during the experiment, three electrodes were placed on the bare upper body of the participant. One lead was placed in the fifth intercostal space on the midclavicular line, another on the manubrium sterni (as a reference), and the last one on the right side of the abdomen (as a ground electrode). The electrodes were connected to a BrainAmp ExG amplifier (Brain Products), and the data were sampled at 1000 Hz using Brain Vision Recorder software. Once the experimenter, located in a room next to the experimental room to oversee the experiment, visually approved the ECG recordings, the experiment began.

The participants received the first three mood questions after they were seated. Once they had answered these questions, they were instructed to press the space bar, and the first image appeared on the screen for 30 s. A new announcement of 5 s then instructed the participants to close their eyes and visualize the image they had just seen. This phase lasted for 1 min. The end of the visualization phase was announced by the experimenter, and new mood questions had to be answered again. The same actions were repeated for the second image. After completing the third and final mood question after seeing and visualizing the second image, the experiment concluded. Besides announcing the end of the experiment and asking whether the participant had recognized one of the two images shown before, the experimenter did not intervene with the participants.

Images were valenced and balanced oppositely, so the first participant was exposed to a positive‒negative sequence and the second participant to a negative‒positive sequence. Another factor that was balanced among participants was presenting them in either colour or black and white. After six months, the experiment was repeated. If a participant was first exposed to a positive-negative sequence, in the second session, they would be exposed to the opposite sequence of images. The same approach was applied for colour or black-and-white images. The study was approved by the medical ethics committee of Maastricht University (NL40284.068.12/METC 12-3-015).

Data are available from the authors upon reasonable request and with permission of Maastricht University.

### 2.4. Data processing

Using the software program Brain Vision Analyzer 2.0, a procedure to automatically detect R-peaks was carried out. The results were visually inspected for incorrectly placed R-peaks. In rare cases where such incorrect R-peaks were detected, the R-peak was manually adjusted or removed. The R-peaks were then transferred to SPSS version 26. Each record represented the time of an R-peak in milliseconds.

This time variable was used to determine heart rate (HR) and heart rate variability (HRV). By summing the time between two successive R-peaks in milliseconds and then dividing this by 60,000, the HR was calculated. To determine the HRV, the square root of the mean absolute differences between RR intervals for each 10-s segment was calculated. This value was then logarithmically transformed to achieve a normal distribution.

These segments, as described before, were built to ensure that the HRV data from all participants contributed equally to the final analyses. For each participant, a total of 18 records were constructed: three for looking at image 1, six for visualizing image 1, three for looking at image 2, and six for visualizing image 2.

The choice for 10-s segments was made to match the intended temporal resolution of the mood induction procedure and to allow repeated within-subject assessment of HRV across the different phases of the experiment. Nevertheless, HRV estimates derived from such ultra-short intervals should be interpreted with caution, as they may be less reliable and less sensitive to dynamic autonomic changes than estimates obtained from longer recordings. This methodological feature is therefore relevant when interpreting the absence of a clear HRV response to the second emotional stimulus.

Finally, the following variables were added to the dataset: a dummy variable that contrasted the second image versus the first image (this variable was set to ‘0’ if participants were first exposed to a negatively valenced image, followed by a positively valenced image, and was set to ‘1’ if participants were exposed to a positively valenced image first, followed by a negatively valenced image), age, sex, colour, and recognition of the images.

The final dataset did not contain data traceable to the subject; subject numbers were used in all files. *P*-values < 0.050 were considered statistically significant.

HRV was the dependent outcome variable in all multi-level analyses. Since the original HRV variable did not meet the criteria for a normal distribution, it was log-transformed. The resulting variable was normally distributed (skewness and kurtosis were 0.656 and 1.599, respectively). Heart rate (HR), the dummy variable, age, sex, colour, and recognition of the image were added as predictors.

To answer the fifth hypothesis, it was necessary to create a second-order interaction. This interaction, formed by the image*dummy variable, was used to determine the effect of the mood switch between the first and the second image on HRV.

## 3. Results

### 3.1. Sample description

Eighty-two participants met the inclusion criteria. There were 27 men (32.9%) and 55 women (67.1%) with a mean age of 41.89 years (SD = 17.02, range 18–65 years).

### 3.2. Descriptive statistics

**Figure 1.**
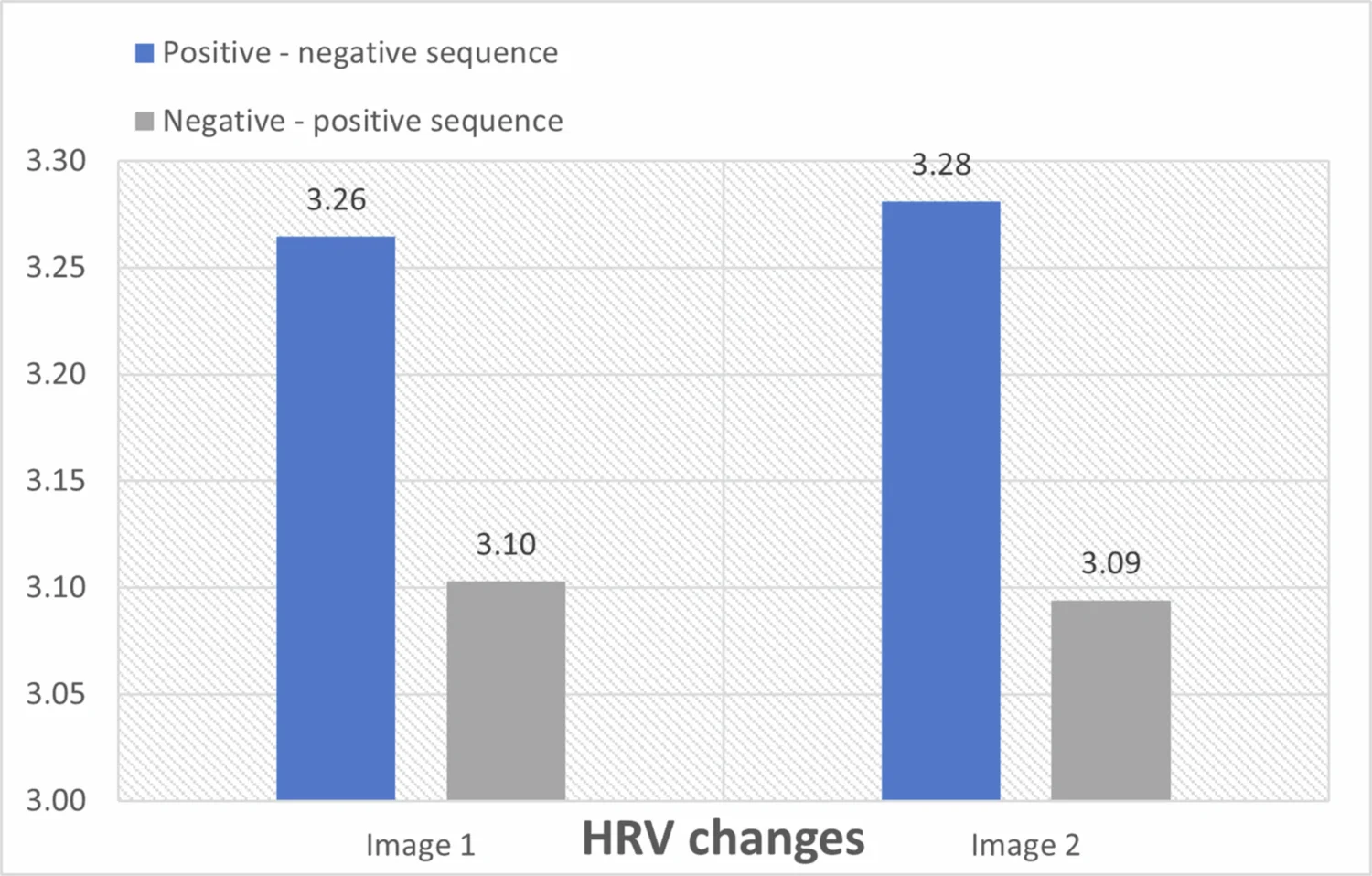
Overview of the HRV levels for images 1 and 2. The blue staves represent the positive‒negative sequence. The grey staves represent the negative-positive sequence.

The changes in HRV between images 1 and 2 are depicted in Figure 1. The blue bars correspond to the change in the HRV level for participants who were exposed to a positively valenced image first and a negatively valenced image second. The grey bars correspond to the change in the HRV level for participants who were exposed to a negatively valenced image first and a positively valenced image second.

First, note the difference between the HRV levels of the two opposite loadings of the first presented image. While a positively valenced image is associated with a higher level of HRV, a negatively valenced image is associated with a lower level of HRV.

Second, the anticipated induction-congruent effect in the HRV from image 1 to image 2 was not observed. Rather, the initial effects of both images on HRV seem to be prolonged during the second image.

Third, there seems to be a trend indicating that the effect from image 1 to image 2 is somewhat greater for those exposed to the positive-negative sequence.

### 3.3. Validating hypotheses

A multi-level regression model was carried out with HRV as the dependent variable and HR, age, and sex as the predictors. Additionally, a random intercept was included, and an AR1 covariance structure was used.

1. Relationship between HR and HRV:

To answer the first hypothesis, HR was tested for significance. The HR variable showed a highly significant effect, indicating that a higher HR is strongly associated with a lower HRV level (estimate −0.015, T = −10.560, and *P* < 0.001), thus confirming the first a priori hypothesis.

2. Relationship between age and HRV:

When examining the age variable, a clear significant effect was also found (estimate −0.009, T = −6.210, and *P* < 0.001). The negative estimate indicates that older participants have a lower level of HRV compared to their younger counterparts.

3. Relationship between sex and HRV:

Although a significant effect on the sex variable emerged (estimate 0.161, T = 3.113, and *P* = 0.003), the effect was in the opposite direction of the a priori expectation: women showed a higher HRV level compared to men.

### 3.4. Post-hoc analysis on the unexpected relationship between sex and HRV

Confronted with this unexpected, highly significant result and based on the results of the earlier-mentioned study by Koenig and Thayer, a new exploratory analysis was carried out: a third-order interaction between HR, age, and sex was tested for significance (Koenig & Thayer, 2016). A marginally significant effect was found (*P* = 0.063). This interaction suggests that older women with higher HR tend to have lower levels of HRV. Consequently, it was decided to incorporate this third-order interaction into further experimental analyses.

### 3.5. Experimental mood induction procedure hypotheses

1. Relationship between mood and the HRV of the first image:

To test the fourth hypothesis, the dummy variable representing the mood character of the first image was tested for significance. For each variable and/or interaction included in the model (see the left column), the results (estimate, T-value, and *P*-value) are presented in Table 1. The mood character variable suggests that when a participant is exposed to a positive image, the HRV level is higher compared to that of a negative image (estimate 0.172, T = 2.885, and *P* = 0.006), thus confirming this hypothesis. Inspecting the covariance parameters, a highly significant AR1 diagonal component was found (*P* < 0.001), symbolizing a strong correlation between adjacent HRV segments and a strong decrease in correlation with segments further apart from one another. Additionally, a highly significant random intercept was found (*P* < 0.001), suggesting that the mean HRV level differs significantly between participants.

2. Relationship between mood and HRV on the second image:

To test the last hypothesis, a second-order interaction between the image variable and the mood character of the image (positive or negative) was tested for significance. A significant interaction effect was found (estimate −0.349, T = −3.481, and *P* = 0.001), as shown in Table 2.

Contrary to the a priori hypothesized effect, this interaction suggests that participants who were exposed to a negatively valenced image first and a positively valenced image second showed a significant decrease in HRV.

### 3.6. Post-hoc analysis on the relationship of the second image and HRV

Confronted with this unexpected result, i.e. a stabilizing or even further proceeding effect of the first image, it was decided to perform further exploratory analyses. In a first post-hoc analysis, the effect of adding the originally reported mood differences (happiness, sadness, and anger) between images 1 and 2 as covariates was investigated. In a second analysis, a third-order interaction between the image, the mood character of the image, and the reported mood was tested for significance. None of these post-hoc analyses showed confounding or interaction effects (all *P*’s > 0.348).

## 4. Discussion

The goal of this study was to investigate whether HRV corresponds with rapidly changing emotions. A previously validated mood induction procedure with two images of opposite emotional loading, presented sequentially to the participants, was carried out while ECG recordings were made. The main finding of this study was that HRV differed in relation to the initial emotional stimulus: participants exposed to an initially positively valenced image exhibited a higher level of HRV compared to those exposed to a first negatively valenced image. However, the HRV did not show the expected reversal after exposure to a second image of opposite emotional loading. Therefore, the present findings support an association between HRV and the initially induced emotional state, but do not provide evidence that HRV dynamically tracks rapid subsequent changes in emotional valence under the current experimental conditions. We will elaborate on these results through several critical points.

A methodological consideration concerns the choice to focus on a single time domain HRV parameter. Given the ultra-short duration of the HRV segments (10 s), only time-domain indices provide reliable estimates; frequency-domain and non-linear HRV measures require substantially longer recordings and do not yield valid or interpretable results in the present design. Time-domain measures seem, therefore, the only robust option for ultra-short HRV estimation. At the same time, the reliance on 10-s HRV segments should be considered an important interpretative constraint. Although these segments allowed the study to examine short-term changes across the mood induction procedure, their reliability and sensitivity may have been insufficient to detect subtle or delayed autonomic responses to the second emotional stimulus. This may partly explain why HRV appeared to reflect the initial emotional induction more clearly than the subsequent change in emotional valence.

With respect to the relationship between HR and HRV, the current literature suggests that as HR increases, HRV decreases (Tsuji et al., 1996). This was also found in the present study. However, another perspective on the association between these two variables is provided by Sacha (Sacha, 2014). Sacha suggested that, in some cases, excluding HR makes HRV a better predictor. He also concludes that when examining HRV values within the same participant, changes in HR should be considered. Since the final dataset from the current study consists of a large number of repeated HRV measurements, HR was added as a predictor to all analyses, in line with Sacha's recommendations.

Second, as stated in the introduction, the relationship between HR, age, and sex is complex (Ryan et al., 1994). These variables are not independent of each other, as illustrated by the significant third-order interaction between HR, age, and sex. Contrary to previous assumptions that lower levels of HRV are typically found in women, our study, along with a large study by Tegegne and colleagues involving 149,205 subjects, found consistently higher HRV in women compared to men (Jensen-Urstad et al., 1997; Tegegne et al., 2018). This finding may initiate a new debate on the relationship between HRV and sex. Additionally, women are historically underrepresented in cardiovascular trials (Cho et al., 2021; Maas et al., 2011). It is suggested that gender differences may impact the ageing process of the heart, and these dependencies tend to diminish with age (Bonnemeier et al., 2003; Olivetti et al., 1995; Voss et al., 2015). A full understanding of gender differences in heart physiology has not yet been achieved. The current literature also suggests emotional response differences between men and women (Bianchin & Angrilli, 2012). Summarizing, a better understanding of the ageing process of the heart, especially in women, combined with the psychophysiological experience of emotions, should be pursued in future research.

Third, it was hypothesized that exposure to a positive image would be associated with a higher level of HRV, and that exposure to a negative image would be associated with a lower level of HRV. The results of the fourth hypothesis confirmed this effect, in line with current evidence that higher HRV is associated with better overall health, while lower HRV is associated with pathological conditions (Tiwari et al., 2021). However, the results of the fifth hypothesis suggested otherwise. Contrary to expectations, the second-order interaction (Image*Mood character) showed a significant negative effect, indicating a lower level of HRV after exposure to a negative-positive sequence of images. When computing the mean HRV levels based on the parameter estimates of the regression model from the fifth hypothesis (Figure 1), the initial effect on HRV (the effect of the first image) persists during exposure to the second image, irrespective of the emotional loading of the first image.

Validation of the mood induction procedure prior to the present study showed a reversal of subjective mood after exposure to the second image, as expected. However, while subjective mood changed congruently with the presented images, the results from the last hypothesis of this study suggest that HRV levels are ‘out of sync’ with reported mood. This asynchrony may indicate that HRV, at least when measured in ultra-short segments in the present sequential design, reflects an initial or sustained autonomic response rather than moment-to-moment changes in subjective emotional valence. One possible explanation could be that the study design is not optimal for observing changing psychophysiological parameters, such as HRV, in rapidly changing emotional states. In particular, the absence of a neutral or washout condition between the two emotionally loaded images may have allowed carryover effects from the first stimulus to persist during the second stimulus. As a result, the autonomic response associated with the first image may have influenced the HRV during the second image, thereby reducing the ability to detect a reversal in the HRV. For further research, it would be interesting to incorporate a neutral image between two emotionally loaded images. It is also possible that the duration of the visualization phase needs to be extended to determine the effect of the second image on the HRV. In summary, more experimental research is needed to develop a ‘rapid changing’ mood induction design to demonstrate covarying subjective mood and HRV effects.

For now, the relationship between rapidly changing emotions and HRV remains unclear. The present findings should therefore not be interpreted as evidence that HRV can track rapid moment-to-moment emotional fluctuations. Rather, they suggest that HRV may be sensitive to an initial emotional stimulus or to a sustained autonomic response following such a stimulus. However, if an adapted experimental study design can demonstrate a closer temporal relationship between subjective reported mood and HRV in the near future, it could have serious clinical implications. After all, if a relationship between subjectively reported mood and HRV is observed, this ‘objectivated and controlled’ mood outcome could be used in psychophysiological research and affective science. Once a better understanding of mood is achieved in healthy individuals, the results might also be translated to clinical populations as well. Since HRV is already known as an indicator of the clinical state in depressed individuals, further evolution of this biomarker could potentially result in better prediction of responses to therapy and recovery (Hartmann et al., 2018). Additionally, HRV may be helpful in exploring differences in the autonomic nervous system between healthy individuals and those with conditions such as schizophrenia and (rapid cycling) bipolar disorder (Faurholt-Jepsen et al., 2017; Henry et al., 2010). Finally, life is a continuous rollercoaster of emotions. Future studies with designs optimized for rapid emotional transitions are needed before HRV can be considered a reliable objective marker of momentary emotional change.

### 4.1. Limitations

There are several limitations of this study that should be mentioned.

The first limitation is the lack of baseline ECG recordings prior to the start of the mood induction procedure. As mentioned before, this study was part of a more extensive study, whose main goal was to investigate psychophysiological reactivity as a result of cognitive, physical, and emotional stressors, during which ECG recordings were made throughout the entire procedure (including a memory task, a stress induction procedure, and a habituation task). However, baseline ECG recordings were made at the beginning of the entire procedure, approximately 1 h before the mood induction procedure started. Therefore, we assumed that these measurements were made too early in the process to create a representative baseline for the participants. Additionally, it did not seem logical to use ECG recordings made during the habituation task, just before the mood induction procedure.

Second, even though the participants were instructed to breathe calmly, no breathing registrations were made; therefore, we were unable to incorporate breathing frequency into the analyses. This is a limitation because incorporating breathing frequency is preferred in research on emotions and HRV (Yamuza et al., 2019). This limitation is particularly relevant in the present study because HRV was estimated using ultra-short 10-s segments. Respiratory variation may have a proportionally greater influence on HRV estimates over such short intervals, and the absence of respiratory measurements may therefore have contributed to uncertainty in the interpretation of the observed HRV patterns, especially the lack of clear responsiveness to the second emotional stimulus.

Third, the exclusion criteria for this experiment did not include a medical history of cardiac problems. However, since the majority of our study group was aged under 50 years, it is unlikely that many subjects in this group had experienced cardiac problems earlier. In the case that some subjects in the remaining group of older participants had experienced cardiac problems, it is unlikely that this outweighed the effects found in the study.

Fourth, the sequential presentation of two emotionally opposite images without a neutral or washout condition may have introduced carryover effects. The HRV response to the first image may have persisted into the second phase of the experiment, thereby limiting the ability to detect whether the HRV changes congruently with the second emotional stimulus. This design constraint should be considered when interpreting the absence of a reversal in the HRV after the second image. Future studies should consider including a neutral interval, a longer recovery period, or a design in which emotional stimuli are presented in separate experimental blocks.

## 5. Conclusions and future directions

Notwithstanding that the present study can be criticized on several points, the findings appear to be valid: when confronted with a first positively valenced image, participants showed a higher level of HRV compared to those exposed to a negatively valenced image. However, the results do not demonstrate that HRV dynamically tracks rapid subsequent changes in emotional valence. Instead, the findings suggest that the HRV may reflect an initial emotional response, or a sustained autonomic response following the first emotional stimulus, under the present experimental conditions. Even though the results for the second image did not confirm the final hypothesis, reasonable post hoc explanations could be provided. Together with a relatively large between-subject sample, a balanced design, and two out of three validating hypotheses confirmed with strong effects, the present study may represent an initial step toward understanding the relationship between the emotional state and HRV, while also showing that the dynamic tracking of rapidly changing emotions by the HRV requires further methodological refinement.

For future research on whether a reversal of HRV effects after exposure to a second image of opposite emotional loading is possible, several potential adjustments to the study design have been proposed. These include the incorporation of a neutral or washout condition, longer recovery or visualization periods, respiratory measurement, and potentially longer HRV recording windows to improve reliability while preserving sensitivity to emotional change. It would also be valuable to explore sex and age-related differences in emotional regulation and HRV more deeply. Replication within clinical populations could further strengthen the relevance of this work. Finally, combining this mood induction procedure with other physiological parameters (e.g. EEG or EMG) may help to further elaborate on the mind-body debate and clarify the observed asynchrony between subjective mood and psychophysiological responses.

Beyond these theoretical implications, the findings also suggest several avenues of practical utility. The sensitivity of the short-term HRV to the initial emotional stimulus indicates that the HRV may serve as a potential marker of initial or sustained emotional responses, rather than as a confirmed marker of moment-to-moment emotional fluctuations. If future studies succeed in demonstrating covariation between rapidly shifting mood states and HRV, this could support the development of real-time psychophysiological monitoring tools. Such tools may ultimately assist in tracking emotional dysregulation, monitoring treatment progress, or identifying early signs of relapse in psychiatric populations. Moreover, the unexpected sex and age-related patterns observed in this study highlight the potential value of more personalized approaches in HRV-based assessment. The present study may therefore provide a basis for the potential clinical and applied relevance of HRV, but only with the important qualification that its usefulness as an objective indicator of rapidly changing momentary emotional state remains to be established.

**Table 1.** The effect of the first image on HRV.

	Dependent variables: HRV, logarithmic			
Variable	Estimate	Std. Error	T-value	*P*-value
Intercept	4.170	0.807	5.166	<0.001
Heart rate	−0.038	0.013	−2.903	0.004
Age	−0.050	0.020	–2.555	0.012
Sex (0 = men, 1 = women)	−2.414	0.876	−2.756	0.007
Age * Sex	0.062	0.021	2.991	0.003
Heart rate * Age	0.001	<0.001	1.923	0.056
Heart rate * Sex	0.038	0.014	2.679	0.008
Heart rate * Age * Sex	−0.001	<0.001	–2.621	0.010
Colour (0 = black and white, 1 = colour)	0.168	0.082	2.053	0.045
Recognition (0 = no, 1 = yes)	−0.015	0.053	−0.280	0.780
Mood character (0 = negative, 1 = positive)	0.172	0.060	2.885	0.006

**Table 2.** The effect of the second image compared to the first image on HRV.

	Dependent variable: HRV, logarithmic			
Variable	Estimate	Std. Error	T-value	*P*-value
Intercept	3.103	0.625	4.963	<0.001
Heart rate	−0.020	0.010	−1.971	0.050
Age	−0.030	0.015	−1.954	0.052
Sex (0 = men, 1 = women)	−1.271	0.676	−1.881	0.061
Age * Sex	0.047	0.016	2.944	0.004
Heart rate * Age	<0.001	<0.001	1.161	0.247
Heart rate * Sex	0.019	0.011	1.802	0.073
Heart rate * Age * Sex	−0.001	<0.001	−2.564	0.011
Colour (0 = black and white, 1 = colour)	0.152	0.069	2.223	0.030
Recognition (0 = no, 1 = yes)	−0.026	0.045	−0.580	0.564
Image (0 = first image, 1 = second image)	0.178	0.051	3.471	0.001
Mood character (0 = negative, 1 = positive)	0.162	0.051	3.145	0.002
Image * Mood character	−0.349	0.100	−3.481	0.001

## Data Availability

The data that support the findings of this study are available from Maastricht University. Restrictions apply to the availability of these data, which were used under license for this study. Data are available from the authors with the permission of Maastricht University.

## Open scholarship

This article has earned the Centre for Open Science badges for Open Materials. The materials are openly accessible at 10.1016/j.xjmad.2023.100002.
